# Supplementary material for: Antibiotic-Induced Treatments Reveal Stress-Responsive Gene Expression in the Endangered Lichen Lobaria pulmonaria
Source: J Fungi (Basel). 2022 Jun 12;8(6):625. doi: 10.3390/jof8060625 (PMC9225190; doi:10.3390/jof8060625)
Supplement: Supplementary file 1 [file jof-08-00625-s001.zip › jof-1751075-supplementary.pdf]

Supplementary Information for

Antibiotic-induced treatments reveal stress-responsive gene expression in the endangered lichen *Lobaria pulmonaria*

Tania Chavarria-Pizarro[1*], Philipp Resl[1,2], Theresa Kuhl [3], Aleksandar Janjic [4], Fernando Fernandez Mendoza[2] and Silke Werth[1]

1. Systematics, Diversity and Evolution of Plants, Faculty of Biology, LMU Munich, Menzingerstraße 67, 80638 Munich, Germany

2. Institute of Biology, University of Graz, Austria

3. Institute for Network Biology (INET). Helmholtz Center Munich, Neuherberg, Germany

4. Anthropology and Human Genomics, Faculty of Biology, LMU Munich, Großhaderner Straße 2-4, 82152 Planegg-Martinsried, Germany

*to whom correspondence should be sent: t.chavarria@lmu.de

This file includes:

Figs. S1-S12

Tables S1-S6

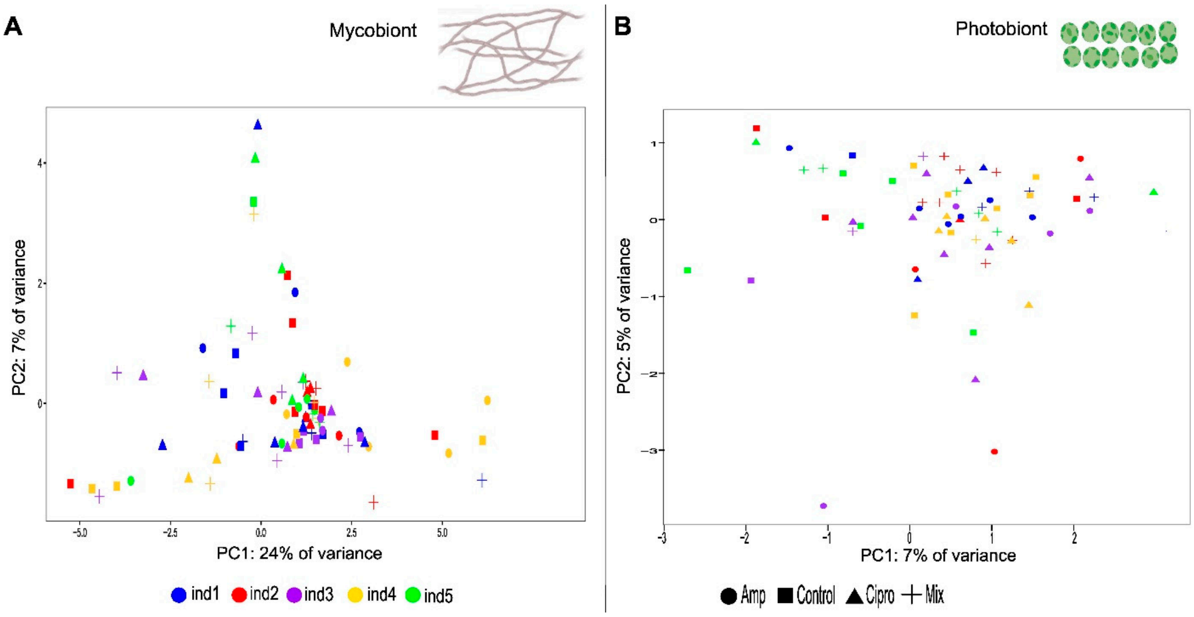

Figure S1. Principal component analysis of gene expression count data for L. pulmonaria included in the antibiotic experiment (see Figure 1). Color-coding represents the different individual thalli used in the experiment: blue (ind1), red (ind2), purple (ind3), yellow (ind4), green (ind5). Symbols represent the different treatments: circle (Ampicillin, Amp), square (Control), cross (Ampicillin + Ciprofloxacin, Mix), triangle (Ciprofloxacin, Cipro). A) Mycobiont B) Photobiont. (n = 96 samples).

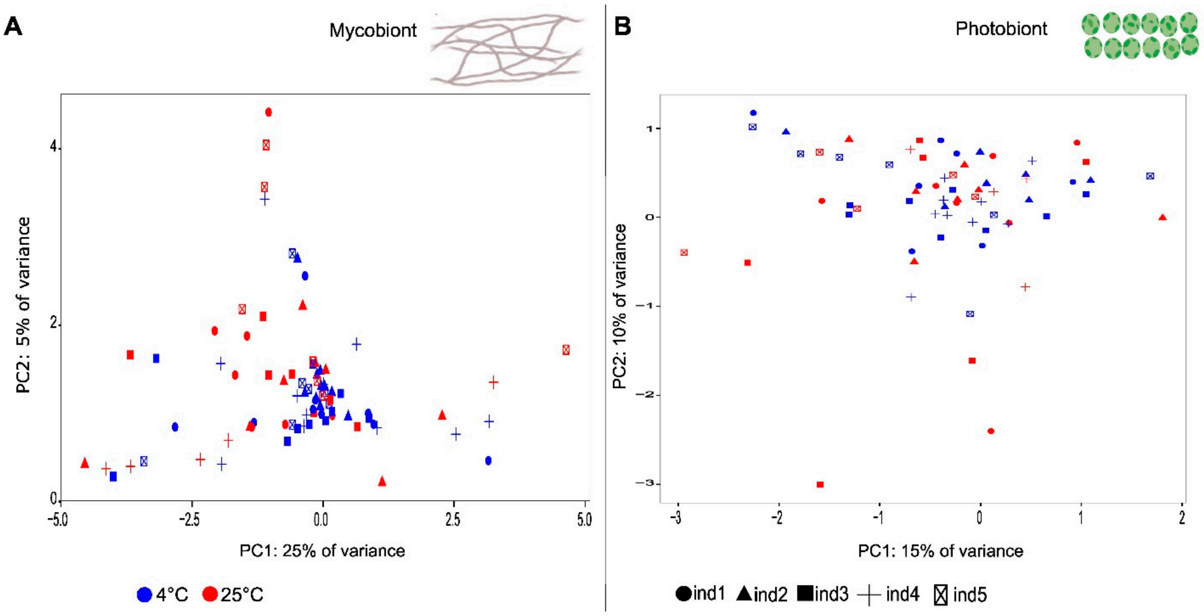

Figure S2. Principal component analysis of gene expression count data for L. pulmonaria included in the antibiotic experiment (see Figure 1). Color-coding represents the temperatures

that the samples were exposed to: red (High temperature, 25 ˚C), and blue (Low temperature, 4 ˚C). Symbols represent the different individual thalli: circle (ind1), triangle (ind2), square (ind3), cross (ind4), square with equis (ind5). A) Mycobiont B) Photobiont. (n = 96 samples).

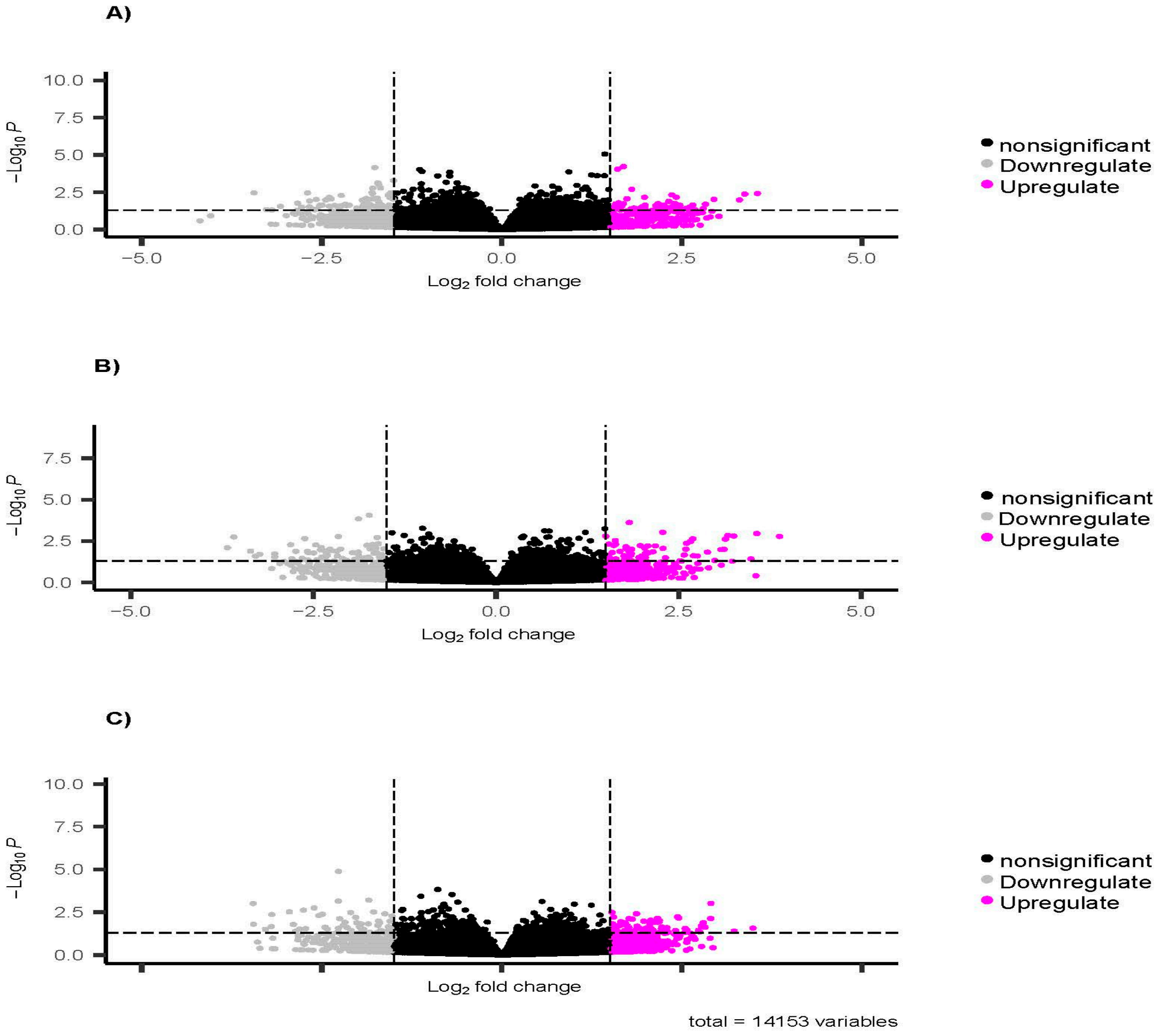

Figure S3. Enhanced volcano plot showing log 2-fold changes and negative log 10 of the probability of differential gene expression in the L. pulmonaria mycobiont between treatments A) Control vs. Ampicillin (Amp) B) Control vs. Ciprofloxacin (Cipro) C) Control vs. Mix (Cipro + Amp). Upregulated genes are in magenta color and downregulated are in grey color. A Benjamini–Hochberg adjusted p-value $p < 0.0001$ was used as a threshold for consideration as DE gene (are above the dashed line); additionally, only transcripts with > 1.5 log2-fold change were considered upregulated and < -1.5 log2-fold change were considered downregulated.

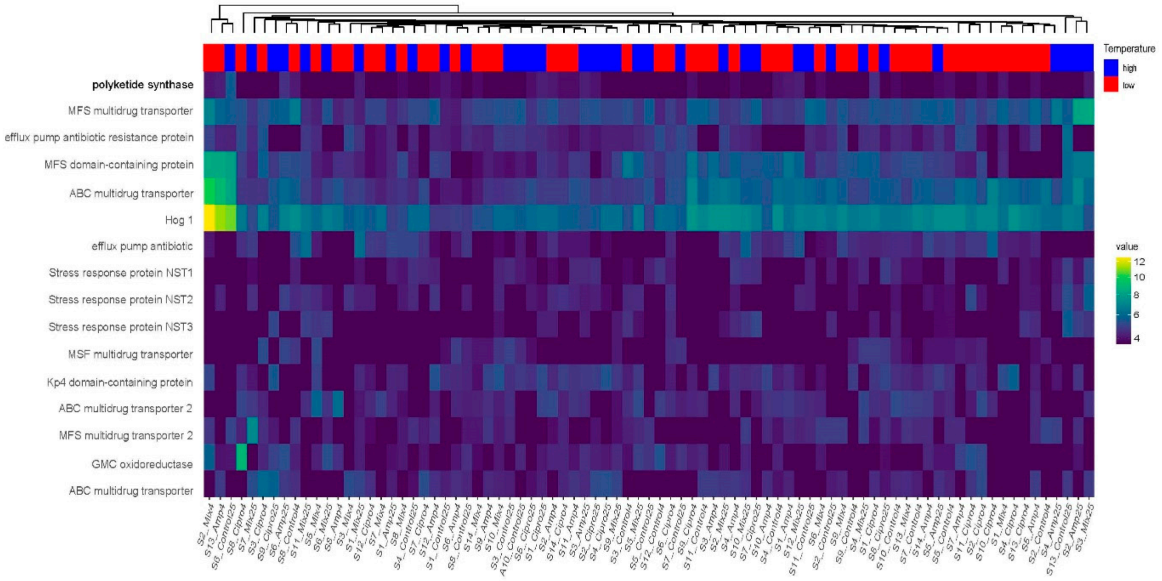

Figure S4. Heatmap showing differentially expressed genes encoding stress-related genes in the mycobiont of the lichen L. pulmonaria at different temperatures: 4 °C (blue), and 25 °C (red). We created the heatmap with the vst-normalized counts and then computed a distance matrix based on the differential expression.

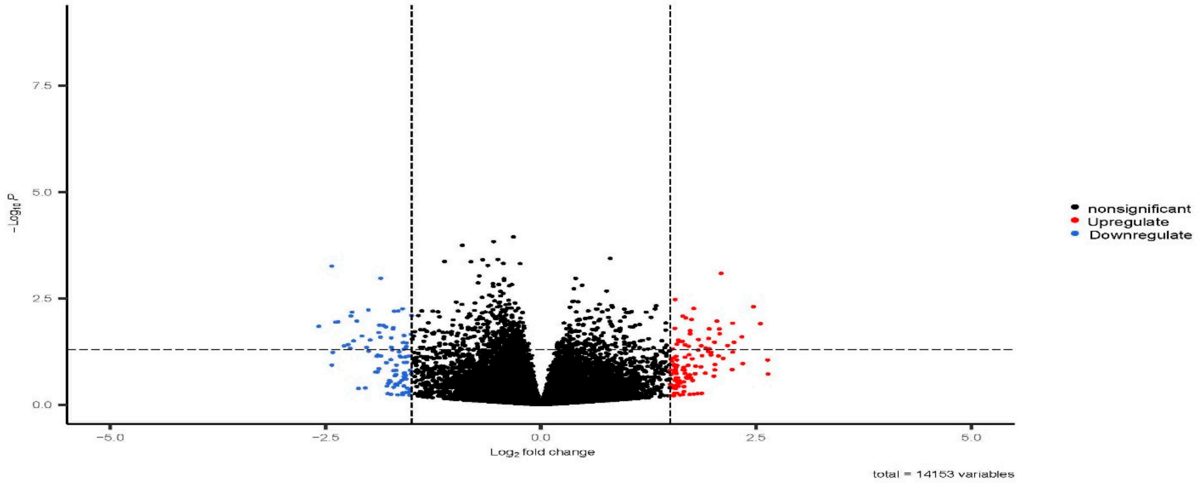

Figure S5. Enhanced volcano plot showing differential gene expression in the mycobiont of L. pulmonaria between high temperatures (25 ˚C) and low temperatures (4 ˚C). Upregulated genes are in red color and downregulated are in blue color. A Benjamini–Hochberg adjusted p-value p < 0.05 was used as a threshold for consideration as DE gene (above the dashed line), additionally only transcripts with > 1.5 log2-fold change were considered upregulated and < -1.5 log2-fold change were considered downregulated.

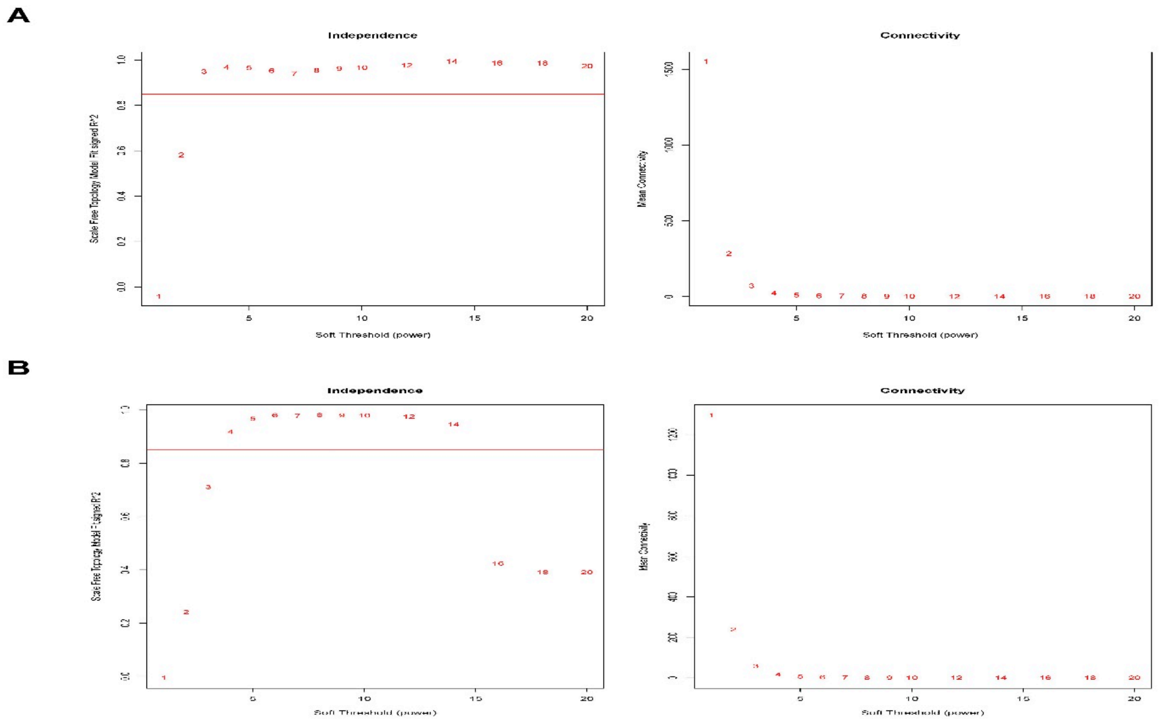

Figure S6. Analysis of network topology for selected soft-thresholding powers. The scale-free fit index as a function of the soft-thresholding power is on the left, and the mean connectivity of the soft-thresholding power is on the right. A) We selected the power 3 for the mycobiont expression set which is the lowest value above 0.85 for which the scale-free topology fits the index curve plateaus until it reaches a high value. B) We selected the power 4 for the photobiont expression set, which is the lowest value above 0.80 for which the scale-free topology fits the index curve plateaus until it reaches a high value.

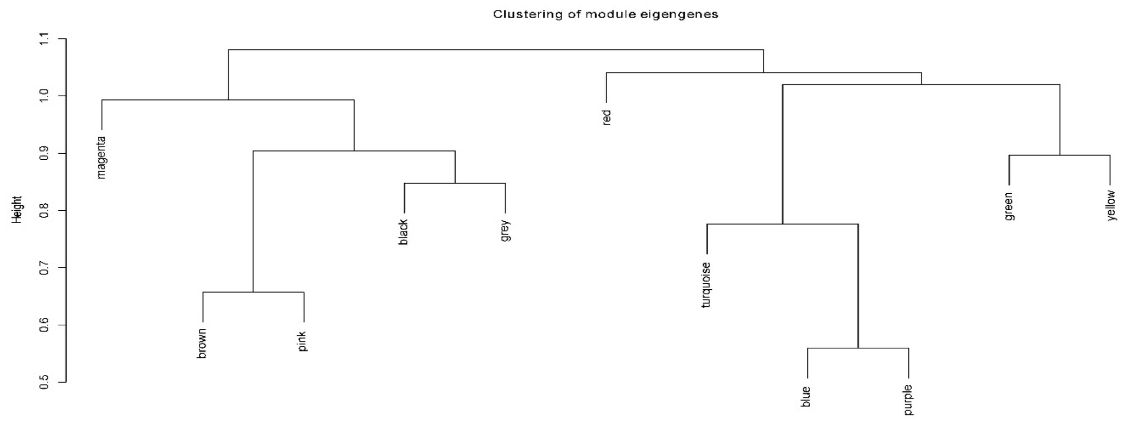

Figure S7. Hierarchical clustering dendrogram of module eigengenes in the L. pulmonaria mycobiont (shown is the first principal component of a module, representative of the overall expression profile for genes within that module).

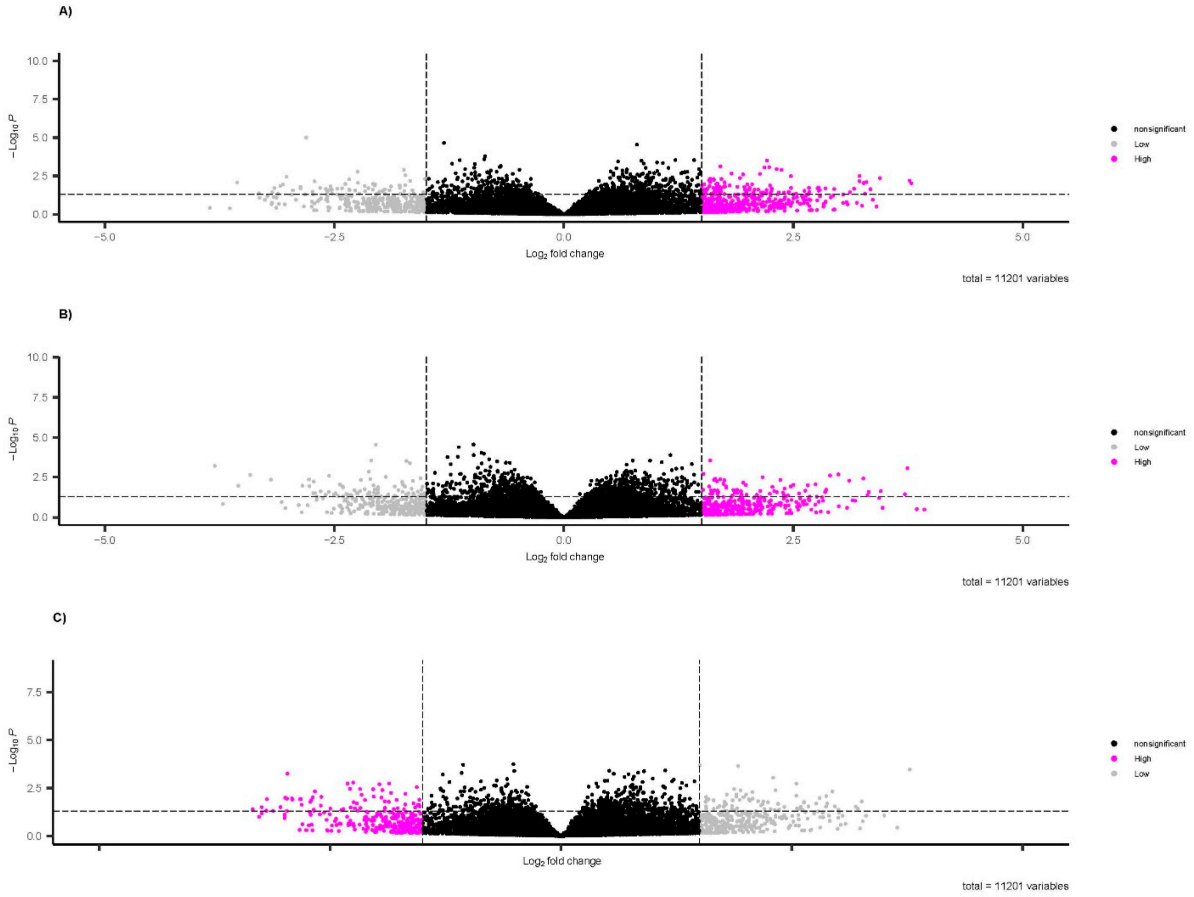

Figure S8. Enhanced volcano plot showing log 2-fold changes and negative log 10 of the probability of differential gene expression in the photobiont S. reticulata between treatments A) Control vs. Ampicillin (Amp) B) Control vs. Ciprofloxacin (Cipro) C) Control vs. Mix (Cipro + Amp). Upregulated genes are in magenta color and downregulated are in grey color. A Benjamini–Hochberg adjusted p-value $p < 0.0001$ was used as a threshold for consideration as DE gene (are above the dashed line); additionally, only transcripts with > 1.5 log2-fold change were considered upregulated and < -1.5 log2-fold change were considered downregulated.

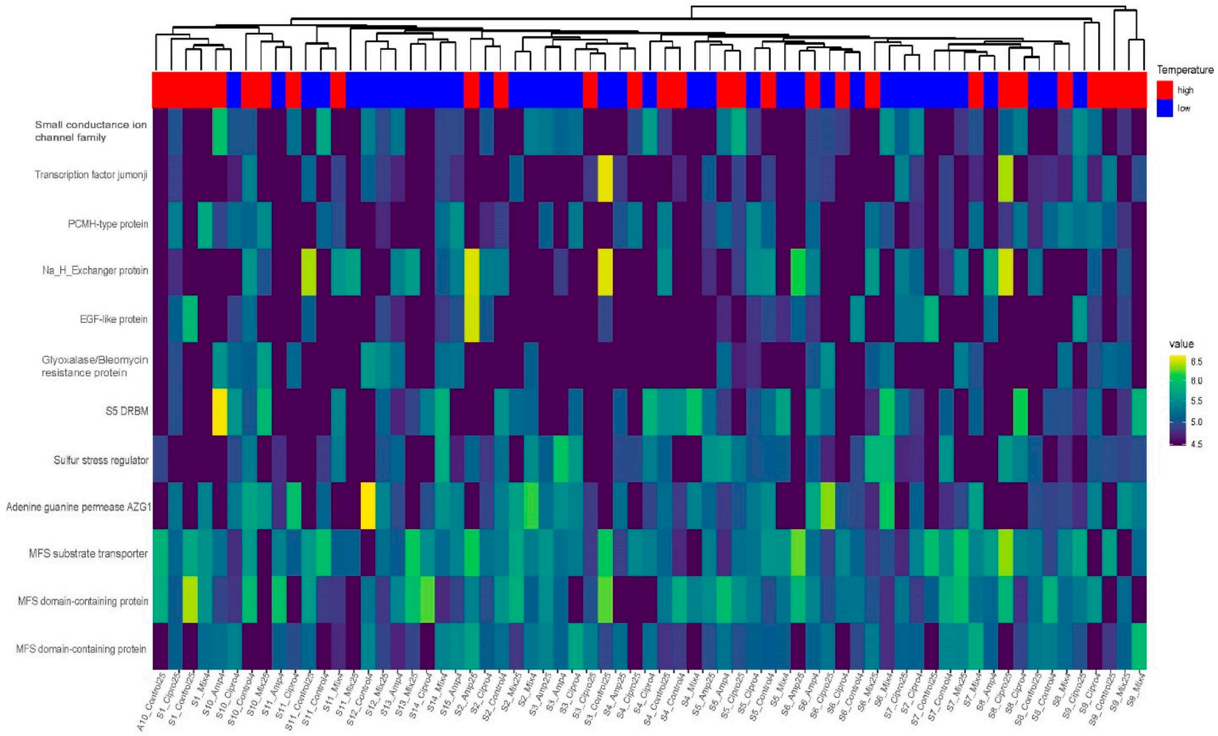

Figure S9. Heatmap showing differentially expressed stress response genes in S. reticulata, the green-algal photobiont of L. pulmonaria, at different temperatures: 4 C˚(blue), 15 C˚(yellow), and 25 C˚(red). We created the heatmap with vst-normalized counts and then by computing a distance matrix based on the differential expression.

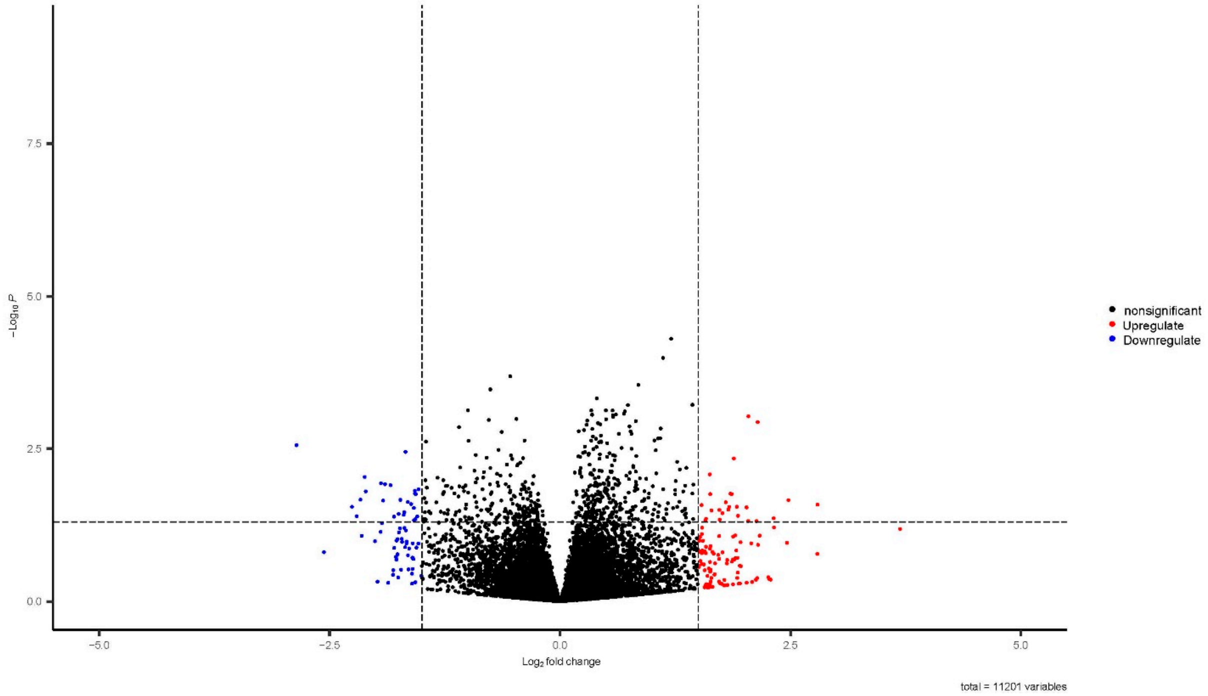

Figure S10. Enhanced volcano plot showing differential gene expression in the photobiont S. reticulata between high temperatures (25 ˚C) and low temperatures (4 ˚C). Upregulated genes are in red color and downregulated are in blue color. A Benjamini–Hochberg adjusted p-value $p < 0.05$ was used as a threshold for consideration as DE gene (above the dashed line); additionally, only transcripts with > 1.5 log2-fold change were considered upregulated and < -1.5 log2-fold change were considered downregulated.

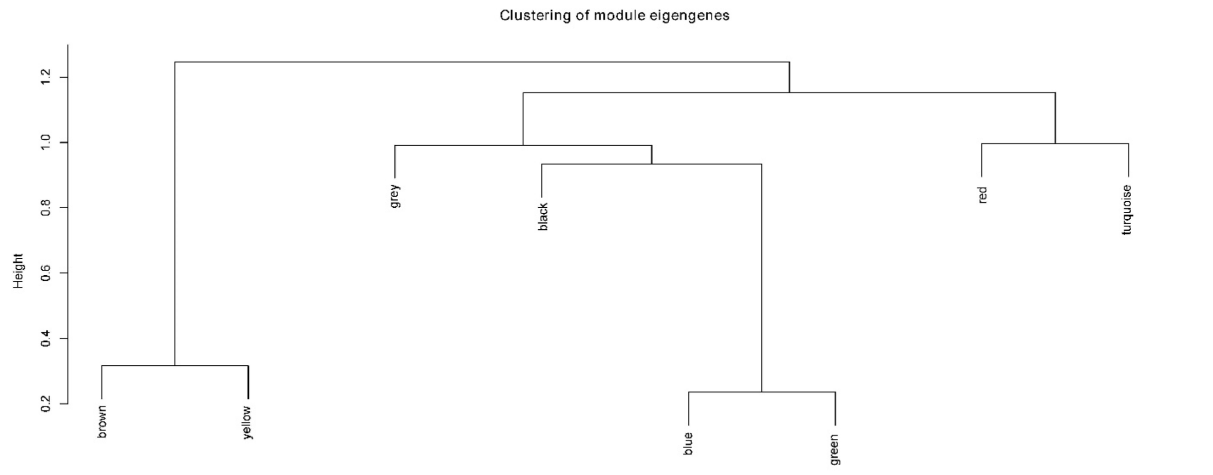

Figure S11. Hierarchical clustering dendrogram of module eigengenes in S. reticulata, the green-algal photobiont of L. pulmonaria (shown is the first principal component of a module, representative of the overall expression profile for genes within that module).

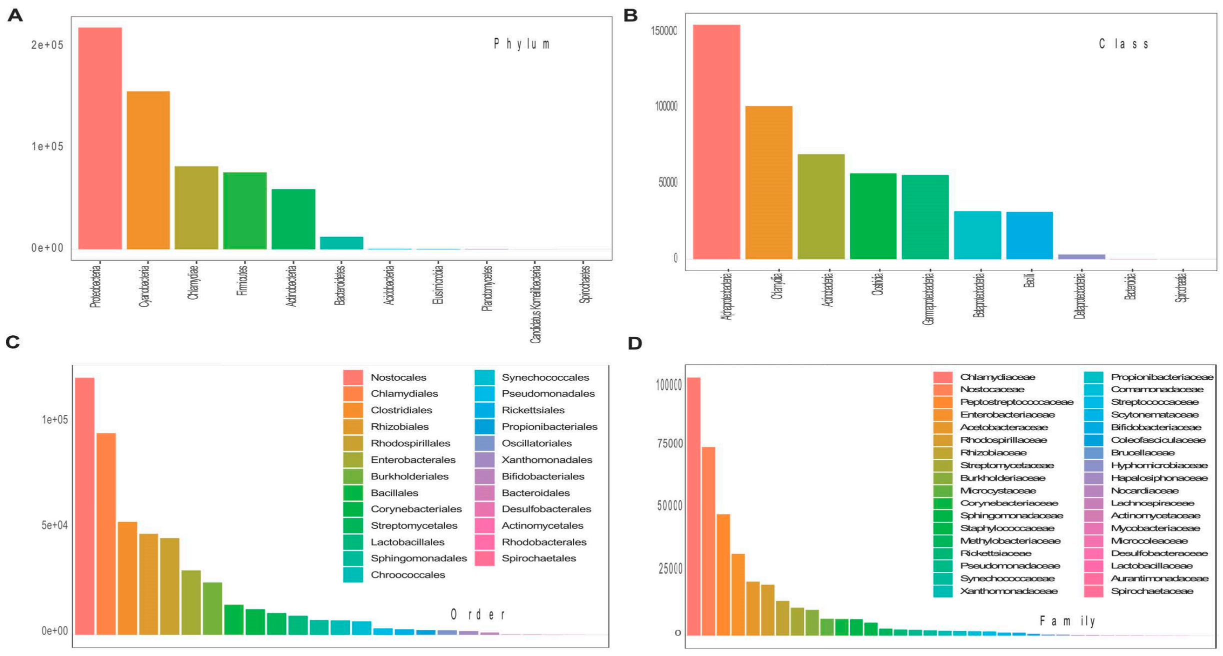

Figure S12. Relative abundance of microbiome in the lichen L. pulmonaria. A) Abundance of bacteria at the phylum level. B) Abundance of bacteria at class level. C) Abundance of bacteria at order level. D) Abundance of bacteria at family level.

Table S1. Differential expression genes in the mycobiont of *L. pulmonaria*. The analysis used pairwise comparisons between samples not treated with Antibiotic (Control), and treated with Antibiotic: Ampicillin (Amp), Ciprofloxaxin (Cipro) and Mix. As threshold values, a log2-fold change >1.5 and Benjamini–Hochberg adjusted p-value <0.05 were used. Genes were identified according to the *L. pulmonaria* genome (JGI) and their function according to the fungi genome database at Uniprot. * target responsive genes.

**Down/Up Regulated**	**Transcript**	**Log2change**	***p*-value**	**Protein**	**Function**
**Amp vs. Control**					
Up at Amp	jgi|Lobpul1|1061842|gw1.190.25.1	2.749458677	0.020386	N-acetyltransferase domain-containing protein	
Up at Amp	jgi|Lobpul1|1076300|e_gw1.39.39.1	31.75542977	0.00486	Cytochrome P450*	Transcription factor
Up at Control	jgi|Lobpul1|1090852|e_gw1.579.26.1	3.147069957	0.00347	BTB domain-containing protein	
Down at Control	jgi|Lobpul1|1093319|e_gw1.739.3.1	-1.690034534	0.71745948	Ubiquitin-associated/translation elongation factor EF1B*	
Up at Amp	jgi|Lobpul1|1097780|e_gw1.1193.5.1	1.458953752	0.028063	Putative oxidoreductase*	
Up at Control	jgi|Lobpul1|1155022|fgenesh1_pg.43_#_17	21.50329367	0.001637	FHA domain-containing protein	
Up at Control	jgi|Lobpul1|1157377|fgenesh1_pg.153_#_18	9.517571688	0.034011	SnoaL-like domain-containing protein	
Down at Amp	jgi|Lobpul1|1157586|fgenesh1_pg.167_#_13	1.327489679	0.047316	ANK_REP_REGION domain-containing protein	
Up at Amp	jgi|Lobpul1|1163545|fgenesh1_pg.745_#_1	3.923271243	0.008724	Actin cytoskeleton-regulatory complex protein PAN2	
Up at Amp	jgi|Lobpul1|1188310|fgenesh1_kg.129_#_87_#_TRINITY_DN11338_c2_g2_i1	3.206864399	0.016926	Dual-specificity kinase *	ATP binding
Up at Control	jgi|Lobpul1|1206444|fgenesh1_kg.332_#_67_#_TRINITY_DN10794_c2_g2_i2	2.281127497	0.026888	GH16 domain-containing protein	
Down at Amp	jgi|Lobpul1|1209203|fgenesh1_kg.369_#_23_#_TRINITY_DN9025_c1_g1_i3			FAD-binding domain-containing protein	
Up at Amp	jgi|Lobpul1|1217968|fgenesh1_kg.523_#_9_#_TRINITY_DN10792_c2_g1_i3	2.646968337	0.039128	Stress response protein NST1*	

Table S1.

Down/Up Regulated	Transcript	Log2change	*p*-value	Protein	Function
Up at Amp	jgi|Lobpul1|1227047|fgenesh1_kg.722_#_12_#_TRINITY_DN11384_c6_g2_i1	1.851	0.042	MFS domain-containing protein*	
Down at Amp	jgi|Lobpul1|1246277|fgenesh1_pm.46_#_3	-1.94	0.030	Delta-12 fatty acid desaturase*	lipid metabolic
Up at Amp	jgi|Lobpul1|1155998|fgenesh1_pg.82_#_20	16.99	5.34E-05	ABC multidrug transporter*	
Up at Amp	jgi|Lobpul1|204752|CE204695_646	1.843	0.031899	ATP dependent ligase	Restored DNA after damage by toxins*
Up at Amp	jgi|Lobpul1|1192049|fgenesh1_kg.165_#_49_#_TRINITY_DN11041_c0_g1_i1	1.62	0.047388	Hog 1*	stress-activated protein kinase osmotic regulation of transcription of target genes *
Up at Amp	jgi|Lobpul1|1260310|gm1.2384_g	1.50	0.035997	ABC multidrug transporter*	
**Cipro vs. Control**					
Down at Cipro	jgi|Lobpul1|1044813|gw1.792.9.1			aspartic-type endopeptidase activity	Peptide metabolism
Up at Control	jgi|Lobpul1|1061842|gw1.190.25.1	2.306086	0.00532	N-acetyltransferase domain-containing protein	
Up at Cipro	jgi|Lobpul1|1076300|e_gw1.39.39.1	2.170438	0.046649	Cytochrome P452*	
Down at Control	jgi|Lobpul1|1090852|e_gw1.579.26.1	-1.402823	0.041544	BTB domain-containing protein	
Up at Cipro	jgi|Lobpul1|1097780|e_gw1.1193.5.1	1.915053	0.03479	Putative oxidoreductase*	
Up at Control	jgi|Lobpul1|1155022|fgenesh1_pg.43_#_17	4.790436	0.016956	FHA domain-containing protein	

Table S1. Continued.

Down/Up Regulated	Transcript	Log2change	*p*-value	Protein	Function
Down at Control	jgi|Lobpul1|1157377|fgenesh1_pg.153_#_18	-2.69304	0.002304	SnoaL-like domain-containing protein	
Down at Cipro	jgi|Lobpul1|1157586|fgenesh1_pg.167_#_13	-2.89306	0.014703	ANK_REP_REGION domain-containing protein	
Up at Control	jgi|Lobpul1|1163545|fgenesh1_pg.745_#_1	2.525057	0.015842	Actin cytoskeleton-regulatory complex protein PAN3	
Up at Cipro	jgi|Lobpul1|1188310|fgenesh1_kg.129_#_87_#_TRINITY_DN11338_c2_g2_i1	3.923271	0.016589	Dual-specificity kinase*	
Down at Control	jgi|Lobpul1|1206444|fgenesh1_kg.332_#_67_#_TRINITY_DN10794_c2_g2_i2	-1.54401	0.00482	GH16 domain-containing protein	
Up at Cipro	jgi|Lobpul1|1217968|fgenesh1_kg.523_#_9_#_TRINITY_DN10792_c2_g1_i3	9.09567	0.001922	Stress response protein NST2*	
Up at Cipro	jgi|Lobpul1|1227047|fgenesh1_kg.722_#_12_#_TRINITY_DN11384_c6_g2_i1	1.271947	0.007961	MFS domain-containing protein*	
Up at Cipro	jgi|Lobpul1|1244062|fgenesh1_kg.1575_#_4_#_TRINITY_DN10677_c0_g3_i1	4.139624	0.001678	Kp4 domain-containing protein*	
Down at Cipro	jgi|Lobpul1|1246277|fgenesh1_pm.46_#_3	-4.037644	0.020267	Delta-12 fatty acid desaturase	
Up at Cipro	jgi|Lobpul1|1073706|e_gw1.8.55.1	2.354399	0.01313	MFS multidrug transporter*	transmembrane transport*
Up at Cipro	jgi|Lobpul1|257964|CE257907_2099	6.422108	0.029438	argininosuccinate synthase	role in cell division, wound healing *
Up at Cipro	jgi|Lobpul1|304760|CE304703_111	3.346162	0.011549	DNA repair protein*	Repair damage to its DNA from environmental *
Up at Cipro	jgi|Lobpul1|1158526|fgenesh1_pg.224_#_11	3.341423	0.035894	efflux pump antibiotic* resistance protein	removes toxic substances, e.g., antimicrobial agents

Table S1. Continued.

Down/Up Regulated	Transcript	Log2change	*p*-value	Protein	Function
Up at Cipro	jgi|Lobpul1|1090417|e_gw1.555.7.1	2.022907	0.032122	ABC multidrug transporter*	
Up at Cipro	jgi|Lobpul1|1073706|e_gw1.8.55.1	1.354598	0.015711	MFS multidrug transporter*	
**Mix vs. Control**					
Up at Mix	jgi|Lobpul1|1089261|e_gw1.487.23.1	2.146947	0.025215	GMC oxidoreductase*	
Down at Mix	jgi|Lobpul1|1155998|fgenesh1_pg.82_#_20	-2.18802	0.011165	Beta-ketoacyl synthase	Fatty acid biosynthesis
Up at Control	jgi|Lobpul1|1157377|fgenesh1_pg.153_#_18	1.721281	0.018564	SnoaL-like domain-containing protein	
Up at Mix	jgi|Lobpul1|1193923|fgenesh1_kg.186_#_4_#_TRINITY_DN11535_c0_g7_i6	3.200919	0.021927	C2H2-type domain-containing protein*	Transcription factor
Down at Mix	jgi|Lobpul1|1209203|fgenesh1_kg.369_#_23_#_TRINITY_DN9025_c1_g1_i3	-1.86918	0.003878	FAD-binding domain	
Up at Mix	jgi|Lobpul1|1217968|fgenesh1_kg.523_#_9_#_TRINITY_DN10792_c2_g1_i3	1.51489	0.025099	Stress response protein NST3*	
Down at Control	jgi|Lobpul1|687803|CE687746_1754	-1.74349	0.019767	Protein kinase domain	
Up at Control	jgi|Lobpul1|913856|CE913799_1780	3.452694	0.00097	SWI/SNF chromatin-remodeling complex	
Up at Control	jgi|Lobpul1|1089261|e_gw1.487.23.1	3.282688	0.031944	GMC oxidoreductase*	
Down at Mix	jgi|Lobpul1|1155998|fgenesh1_pg.82_#_20	-1.65662	0.017819	Beta-ketoacyl synthase	Fatty acid biosynthesis
Up at Control	jgi|Lobpul1|1157377|fgenesh1_pg.153_#_18	2.754345	0.002395	SnoaL-like domain	
Up at Mix	jgi|Lobpul1|1193923|fgenesh1_kg.186_#_4_#_TRINITY_DN11535_c0_g7_i6	2.028076	0.006257	C2H2-type domain*	Transcription factor
Down at Mix	jgi|Lobpul1|1209203|fgenesh1_kg.369_#_23_#_TRINITY_DN9025_c1_g1_i3	-1.55105	0.006034	FAD-binding domain	
Up at Mix	jgi|Lobpul1|1219487|fgenesh1_kg.551_#_21_#_TRINITY_DN11544_c5_g9_i1	1.779607	0.003042	efflux pump antibiotic*	
Up at Mix	jgi|Lobpul1|1163408|fgenesh1_pg.723_#_2	1.542306	0.028294	polyketide synthase*	antimicrobial and immunosuppressive *

Table S2. Differential expression genes in the mycobiont of *L. pulmonaria*. The analysis used pairwise comparisons between samples treated with high temperature (25 ˚C) and low temperature (4 ˚C). As threshold values, a log2-fold change >1.5 and Benjamini–Hochberg adjusted p-value <0.05 were used. Genes were identified according to the *L. pulmonaria* genome (JGI) and their function according to the fungi genome database at Uniprot.

**Down/Up Regulated**	**Transcript**	**Log2change**	***p*-value**	**Protein**	**Function**
**25 °C vs. 4 °C**					
Down at 25	jgi|Lobpul1|1045812|gw1.238.4.1	-1.59968	0.034417	ANK_REP_REGION domain-containing protein	
Up at 25	jgi|Lobpul1|1051947|gw1.387.10.1	1.240632339	0.025148	HET domain-containing protein	
Down at 4	jgi|Lobpul1|1060469|gw1.374.11.1	-1.56265	0.042805	HET domain-containing protein	
Up at 25	jgi|Lobpul1|1075956|e_gw1.33.82.1	1.5105086	0.012035	protein kinase	transfer phosphate, ATP
Up at 25	jgi|Lobpul1|1081549|e_gw1.177.43.1	3.48046219	0.029996	MAP kinase SskB*	Response to heat, osmotic stress*
Down at 25	jgi|Lobpul1|1078849|e_gw1.96.23.1	-1.88187	0.019992	TPR_REGION domain-containing protein	
Down at 4	jgi|Lobpul1|1099469|e_gw1.1509.2.1	-2.17442	0.031321	PNP_UDP_1 domain-containing protein	
Down at 4	jgi|Lobpul1|1100190|estExt_Genewise1Plus.C_10004	-1.60657	0.005508	Putative GNAT acetyltransferase	
Down at 25	jgi|Lobpul1|1110751|estExt_Genewise1Plus.C_3310026	-1.85978	0.001057	DUF676 domain-containing protein	
Up at 4	jgi|Lobpul1|1138430|estExt_Genewise1.C_5690016	1.71	0.046	ANK_REP_REGION domain-containing protein	
Down at 25	jgi|Lobpul1|1156388|fgenesh1_pg.101_#_14	-1.70287	0.006287	ANK_REP_REGION domain-containing protein	
Down at 4	jgi|Lobpul1|1157602|fgenesh1_pg.168_#_15	-2.57941	0.014264	Kinesin motor domain-containing protein	
Down at 4	jgi|Lobpul1|1159332|fgenesh1_pg.281_#_4	-1.5087	0.021952	Urea active transporter	
Down at 4	jgi|Lobpul1|1160352|fgenesh1_pg.365_#_6	-1.59123	0.049	ANK_REP_REGION domain-containing protein	

Table S2. Continued

Down/Up Regulated	Transcript	Log2change	*p*-value	Protein	Function
Up at 25	jgi|Lobpul1|1161527|fgenesh1_pg.478_#_11	1.69	0.0327	HET domain-protein	
Up at 4	jgi|Lobpul1|1220557|fgenesh1_kg.572_#_25_#_TRINITY_ DN10504_c0_g1_i1	1.024	0.0338	Chitin-binding type-1 domain-containing protein	
Down at 25	jgi|Lobpul1|1259647|gm1.1721_g	-2.21	0.0470	Glycosyl hydrolase	
Up at 25	jgi|Lobpul1|1175972|fgenesh1_kg.39_#_49_#_TRINITY_ DN9593_c1_g2_i2	1.70	0.0164	3-hydroxyisobutyrate dehydrogenase	Reduction NAP to NAPH
Up at 25	jgi|Lobpul1|1186473|fgenesh1_kg.114_#_164_#_TRINITY _DN11600_c3_g12_i1	1.27	0.0049	branched-chain amino acid aminotransferase	Catalysis biochemical rx physiological temperatures
Up at 25	jgi|Lobpul1|1259869|gm1.1943_g	1.73	0.0098	branched-chain amino acid aminotransferase	Catalysis biochemical rx physiological temperatures
Up at 25	jgi|Lobpul1|1266787|gm1.8861_g	1.47	0.0376	disulfide isomerase*	maintains the redox environment of a cell exposes to stress *
Up at 25	jgi|Lobpul1|1269925|gm1.11999_g	5.84	0.0090	cytochrome P450*	Catalysis of an oxidation-reduction (redox) reaction
Up at 25	jgi|Lobpul1|555700|CE555643_29333	3.31	0.0159	MFS lactose permease*	Transmembrane transport
Up at 4	jgi|Lobpul1|756045|CE755988_2676	1.93	0.0212	allantoate permease*	Transmembrane transport
Down at 4	jgi|Lobpul1|102832|CE102775_1579	-1.57	0.0462	WetA developmental regulatory protein	producing non-motile spores, via mitotic asexual reproduction in fungi
Down at 15	jgi|Lobpul1|1038709|gw1.7.2.1	-2.00	0.0058	NRPS-like enzyme*	Carbohydrate metabolic process*
Down at 4	jgi|Lobpul1|1080098|e_gw1.129.23.1	-2.13	0.0107	dimethylaniline monooxygenase	Chemical rx and pathways by which a cell derives energy
Down at 25	jgi|Lobpul1|1095268|e_gw1.899.8.1	-2.28	0.0411	palmitoyltransferase	Metabolism lipids
Down at 25	jgi|Lobpul1|1217558|fgenesh1_kg.514_#_61_#_TRINITY_ DN11473_c1_g13_i1	-1.72	0.0163	zinc finger protein DHHC	Metabolism lipids

Table S2. Continued.

**Down/Up Regulated**	**Transcript**	**Log2change**	***p*-value**	**Protein**	**Function**
Down at 25	jgi|Lobpul1|220108|CE220051_1898	-2.35513	0.011142	karyopherin alpha subunit	Regulatory stages of asexual and sexual development.
Up at 4	jgi|Lobpul1|1261443|gm1.3517_g	1.534850276	0.017929	N-acetyltransferase protein	
Down at 4	jgi|Lobpul1|1266617|gm1.8691_g	-2.42777	0.000547	Helo_like_N protein	
Up at 4	jgi|Lobpul1|1270058|gm1.12132_g	1.909721863	0.01073	Magnesium transport protein CorA,	Transmembrane transport
Up at 4	jgi|Lobpul1|1270289|gm1.12363_g	0.592657098	0.040187	Protein kinase domain-containing protein	
Down at 4	jgi|Lobpul1|1272499|gm1.14573_g	-2.02266	0.04457	SMP domain-containing protein	
Down at 25	jgi|Lobpul1|179989|CE179932_381	-1.86166	0.025703	ANK_REP_REGION domain-containing protein	
Down at 25	jgi|Lobpul1|309425|CE309368_466	-2.23643	0.038242	ANK_REP_REGION domain-containing protein	
Up at 4	jgi|Lobpul1|560648|CE560591_49629	3.60	0.032257	F-box domain-containing protein	
Down at 4	jgi|Lobpul1|894361|CE894304_2031	-1.71603	0.016341	Vacuolar-protein-sorting/targeting protein 10	Protein transport

Table S3. Selected GO term results from functional enrichment analyses of genes within WGCNA module eigengenes mycobiont.

Transcript	Go ID	Go Name	Go category	goAcc
Grey Antibiotic Temperature R^2= -0.25				
479713	16	mitochondrial genome maintenance	biological_process	GO:0000002
479713	257	mitochondrial chromosome	cellular_component	GO:0000262
1165599	23919	FAD binding	molecular_function	GO:0050660
1165599	2290	catalytic activity	molecular_function	GO:0003824
1241701	2888	monooxygenase activity	molecular_function	GO:0004497
1241701	10964	salicylate 1-monooxygenase activity	molecular_function	GO:0018658
1241701	4851	aromatic compound metabolic process	biological_process	GO:0006725
1241701	5830	metabolic process	biological_process	GO:0008152
1241701	4283	electron transport	biological_process	GO:0006118
1268438	3200	ubiquitin-protein ligase activity	molecular_function	GO:0004842
1268438	24691	negative regulation of ubiquitin-protein ligase activity	biological_process	GO:0051444
1268438	24685	regulation of ubiquitin-protein ligase activity	biological_process	GO:0051438
1268438	5939	zinc ion binding	molecular_function	GO:0008270
1268438	24687	regulation of ubiquitin-protein ligase activity during meiotic cell cycle	biological_process	GO:0051440
1268438	24690	positive regulation of ubiquitin-protein ligase activity	biological_process	GO:0051443
1326401	5830	metabolic process	biological_process	GO:0008152
1326401	9710	oxidoreductase activity	molecular_function	GO:0016491
Yellow Antibiotic Treatment R^2= -0.23				
766509	4143	carbohydrate metabolic process	biological_process	GO:0005975
766509	10063	intramolecular transferase activity, phosphotransferases	molecular_function	GO:0016868
766509	2984	phosphoacetylglucosamine mutase activity	molecular_function	GO:0004610
Turquoise Antibiotic treatment R^2= 0.59				
1263640	9320	membrane	cellular_component	GO:0016020
1263640	8535	amino acid transmembrane transporter activity	molecular_function	GO:0015171
1263640	4977	amino acid transport	biological_process	GO:0006865

Table S4. Differential expression genes in *Symbiochloris reticulata*, the green-algal photobiont of the lichen *L. pulmonaria*. The analysis used pairwise comparisons between samples not treated with Antibiotic (Control), and treated with Antibiotic: Ampicillin (Amp), Ciprofloxaxin (Cipro) and Mix,with a log2-fold change >1.5 and Benjamini–Hochberg adjusted p-value <0.05. Genes were identified according to the *S. reticulata* genome annotation from JGI and their function was derived by homology to the plant genome database at Uniprot.

**Down/Up regulated**	**Transcript**	**Log2change**	***p*-value**	**Protein**	**Function**
**Amp vs. Control**					
Down at Amp	584	-2.238	0.010543	SET domain-containing protein	methylation
Up at Control	896	3.219838	0.006884	WD_REPEATS_REGION domain-containing protein	
Down at Control	1014	-2.24666	0.001694	MYB transcription factor*	DNA biding
Down at Amp	1210	-2.12971	0.019874	Squalene synthase	Lipid biosynthesis
Up at Control	1253	1.951078	0.002494	Lysophosphatidic acid phosphatase type 6	
Up at Amp	1383	2.264727	0.021287	Cytochrome P450*	
Down at Amp	2708	-1.72581	0.466834	Putative extracellular protein CSOL_018	methylation
Down at Control	2713	-2.80654	0.106592	Argininosuccinate lyase	
Down at Amp	584	-2.04006	0.75147	SET domain-containing protein	DNA biding
Up at Amp	3066	3.115507	0.036508	CLP1_P domain-containing protein*	ATP binding
Down at Control	3807	-3.06058	0.624937	Reverse transcriptase	
Down at Control	4243	-1.77964	0.751218	Amidase signature enzyme	
Up at Amp	4362	1.697388	0.049864	Glyoxalase/Bleomycin resistance protein/Dihydroxybiphenyl dioxygenase*	Redox
Up at control	4901	1.755686	0.015267	EGF-like domain-containing protein	
Down at Control	5584	-2.5541	0.026253	Glycosyltransferase family 92 protein	

Table S4. Continued.

Down/Up regulated	Transcript	Log2change	*p*-value	Protein	Function
Up at Amp	6345	2.20	0.0438	Agenet-like domain-containing protein*	transcription initiation
Down at Amp	6378	-3.17671	0.036915	DAD1	
Down at Control	6449	-1.97421	0.032417	Flavin-containing monooxygenase*	NAP binding
Up at Control	7285	1.826572	0.002198	Plasma membrane ATPase	
Down at Control	7526	-1.8	0.027	Gamma-tubulin complex component 2	
Up at Control	7654	2.776076	0.024696	Protein serine/threonine phosphatase 2C	
Up at Control	7754	1.973538	0.025413	Serine threonine-kinase SRK2E-like	
Down at Amp	7759	-1.78739	0.01834	Methyl-accepting transducer domain-containing protein	
Up at Amp	7602	2.662416	0.047813	FAD-binding PCMH-type protein*	oxidoreductase activity
Up at Amp	9239	1.74894	0.047767	Small-conductance mechanosensitive ion channel family *	
Down at Amp	9256	-1.64939	0.027314	Small ubiquitin-related modifier	
Up at Control	9472	2.172812	0.042341	USP domain-containing protein	
Up at Control	9639	3.443647	0.004403	Non-specific serine/threonine kinase	
Down at Conreol	9686	-2.95122	0.027403	Dihydrodipicolinate reductase chloroplastic-like	
Up at Amp	9958	1.671791	0.023621	E3 ubiquitin protein ligase	protein modification
Up at Amp	10081	1.711246	0.020326	Exostosin-like glycosyltransferase	
Up at Control	10408	1.716358	0.005034	WD40 repeat-like protein	
Up at Amp	10748	1.804726	0.0132	Hexosyltransferase	protein glycosylation
Up at Amp	11397	2.672953	0.035013	MFS domain-containing protein*	transmembrane transporter

Table S4. Continued.

Down/Up regulated	Transcript	Log2change	*p*-value	Protein	Function
Down at Amp	11146	-2.57616	0.027855	Methyltransferase sll0829	
Up at Control	11256	3.162374	0.021374	SMP-LTD domain-containing protein	lipid transport
Up at Amp	11345	2.244906	0.01327	PseudoU_synth_2 domain-containing	RNA biding
Up at Amp	11607	2.050505	0.011149	MFS domain-containing protein*	
Up at Control	12711	2.944593	0.022457	Gluconokinase	
**Cipro vs. Control**					
Up at Control	152	1.89	0.04013	S5 DRBM domain-containing protein	translation
Down at Cipro	949	-2.55878	0.002652	Cellulase domain-containing protein	Carbohydrate metabolism
Down at Control	3203	-2.0367	0.034769	Sucrose-phosphatase	
Down at Control	3725	-3.41731	0.002289	BED-type domain-containing protein	DNA biding
Down at Cipro	3756	-1.71059	0.04335	protein folding	
Down at Cipro	5736	-1.63378	0.040053	1-(5-phosphoribosyl)-5-[(5-phosphoribosylamino) methylideneamino] imidazole-4-carboxamide isomerase	Chloroplastic protein
Up at Control	6352	2.28235	0.020451	Enkurin domain-containing protein	
Down at Control	6838	-2.41059	0.012036	Thioredoxin-dependent peroxiredoxin	
Down at Cipro	6961	-2.77236	0.011312	DnaJ-domain-containing protein	

Table S4. Continued.

Down/Up regulated	Transcript	Log2change	*p*-value	Protein	Function
Up at Cipro	8777	2.470173	0.011974	Transcription factor jumonji*	
Up at Control	10184	1.519255	0.001983	DUF221-domain-containing protein	
Up Cipro	10661	1.74786	0.004925	Armadillo repeat-containing protein 8	
**Mix vs. Control**					
Down at Control	268	-3.19103	0.047718	Replication factor C subunit 3-like	DNA replication
Down at Control	297	-2.15942	0.007133	Rieske domain-containing protein	Nitrate assimilation
Up at Mix	1481	1.853307	0.02733	Sulfur stress regulator*	protein kinase activity
Down at Control	2024	-1.91354	0.048908	L-amino-acid oxidase*	
Down at Mix	2518	-2.59486	0.009147	Ceramide glucosyltransferase	
Up at Control	2631	1.095385	0.030214	Xylose isomerase protein	
Up at Mix	2954	1.925634	0.044702	FAD-binding PCMH-type protein*	oxidoreductase activity
Down at Mix	3703	-1.53576	0.024386	Prefoldin beta-like protein	
Up at Control	4424	2.323134	0.00409	NDC10_II domain-containing protein	
Up at Mix	4739	2.556206	0.035811	J domain-containing protein	
Up at Mix	5241	3.005322	0.042539	LNS2 domain-containing protein	
Up at Control	5362	3.217045	0.036963	Protein kinase domain protein	
Down at Control	5548	-1.53321	0.012768	Lactamase_B domain-containing protein	
Up at Mix	5578	1.587247	0.046877	N-terminal nucleophile aminohydrolase	

Table S4. Continued.

**Down/Up regulated**	**Transcript**	**Log2change**	***p*-value**	**Protein**	**Function**
Up Control	6201	1.51826	0.044273	PPM-type phosphatase protein	
Down at Mix	6409	-1.76804	0.035106	Flavin-containing monooxygenase	
Up at Control	6733	5.487569	0.005174	STAS domain-containing protein	
Up at Mix	6775	3.430975	0.013289	tRNA-uridine amino carboxy propil transferase	
Up at Mix	7142	5.422218	0.000224	Na_H_Exchanger domain protein	
Up at Mix	8293	3.254776	0.021797	Sulfurtransferase	
Down at Control	9282	-2.66395	0.004784	Aa_trans domain-containing protein	
Up at Mix	9949	3.254776	0.021797	Kinase-like protein	
Up at Mix	1383	4.706881	0.016497	Adenine guanine permease AZG1*	Membrane transporter
Down at Mix	10838	-1.93365	0.004042	TPT domain-containing protein	
Down at Control	11657	-2.6786	0.042358	Variable flagella 1	
Down at Control	12347	-2.81526	0.011933	MFS general substrate transporter*	
Down at Mix	11992	-3.03748	0.031075	Succinate dehydrogenase	
Up at Control	12377	3.298754	0.025245	Nucleolar 14-like	ribosome biogenesis
Down at Control	12347	-1.56166	0.002856	Hyperpolarization activated cyclicnucleotide-gated potassium channel 4 *	

Table S5. Differential expression genes in *Symbiochloris reticulata*, the green-algal photobiont of the lichen *L. pulmonaria*. The analysis used pairwise comparisons between samples treated with high temperature (25 ˚C) and low temperature (4 ˚C), with a log2-fold change >1.5 and Benjamini–Hochberg adjusted p-value <0.05. Genes were identified according to the *S. reticulata* genome annotation from JGI and their function was derived by homology to the plant genome database at Uniprot.

**Down/Up regulated**	**Transcript**	**Log2change**	***p*-value**	**Protein**	**Function**
Down at 4	508	-2.14	0.034	Alpha beta hydrolase	
Down at 25	3173	-1.98	0.039	TPT-domain-containing protein	
Down at 25	3711	-2.31	0.023	Nicotinate phosphoribosyltransferase	
Up at 4	5893	2.34	0.026	N-terminal nucleophile aminohydrolase	
Down at 25	7371	-1.58	0.030	CBFD_NFYB_HMF domain-containing protein	
Down at 4	7864	-3.10	0.020	5'-deoxynucleotidase	
Up at 25	7906	2.76	0.032	DNA ligase	
Up at 25	10900	1.73	0.028	Cytochrome P450	

Table S6. Select GO term results from functional enrichment analyses of genes within WGCNA module eigengenes photobiont.

**Transcript**	**Go ID**	**Go Name**	**Go category**	**goAcc**
Turquoise Antibiotic treatment R^2= 0.22				
1707	10013	hydrolase activity	molecular_function	GO:0016818
1707	3736	ATP binding	molecular_function	GO:0005524
3391	4602	protein amino acid phosphorylation	biological_process	GO:0006468
3391	3736	ATP binding	molecular_function	GO:0005524
3391	3043	protein kinase activity	molecular_function	GO:0004672
3997	111	imidazoleglycerol-phosphate synthase activity	molecular_function	GO:0000107
3997	10461	NAD(P)-asparagine ADP-ribosyltransferase activity	molecular_function	GO:0018121
3997	10467	NAD(P)-serine ADP-ribosyltransferase activity	molecular_function	GO:0018127
3997	19331	7-cyano-7-deazaguanine tRNA-ribosyltransferase activity	molecular_function	GO:0043867
3997	10420	NAD(P)-cysteine ADP-ribosyltransferase activity	molecular_function	GO:0018071
8744	10269	nucleoside-triphosphatase activity	molecular_function	GO:0017111
8744	2176	DNA binding	molecular_function	GO:0003677
8744	3838	nucleus	cellular_component	GO:0005634
8744	3736	ATP binding	molecular_function	GO:0005524
8744	162	nucleotide binding	molecular_function	GO:0000166
8744	3862	DNA replication factor C complex	cellular_component	GO:0005663
8744	4421	DNA replication	biological_process	GO:0006260
11957	5939	zinc ion binding	molecular_function	GO:0008270
11957	3727	protein binding	molecular_function	GO:0005515
8744	10269	nucleoside-triphosphatase activity	molecular_function	GO:0017111
